# Giant Gastrointestinal Stromal Tumor Presenting as a Palpable Abdominal Mass: An Unusual Presentation

**DOI:** 10.5402/2011/894829

**Published:** 2011-06-16

**Authors:** Sachin Patil, Sudhir Jain, R. C. M. Kaza, Ronald S. Chamberlain

**Affiliations:** ^1^Department of Surgery, Saint Barnabas Medical Center, Livingston, NJ 07039, USA; ^2^Department of Surgery, Maulana Azad Medical College, New Delhi 110002, India; ^3^Saint George's University School of Medicine, Grenada, West Indies; ^4^Department of Surgery, University of Medicine and Dentistry of NJ (UMDNJ), Newark, NJ 07039, USA

## Abstract

Gastrointestinal stromal tumors (GIST-) account for the majority of mesenchymal tumors arising within the gastrointestinal tract. GIST presenting as a palpable abdominal mass is extremely rare. We report four additional cases of a GIST presenting as an abdominal mass along with a pertinent review of the literature. Twenty five cases of GISTs presenting with an abdominal mass, including 4 cases discussed here, have been reported in the world literature since 2001. The mean duration of symptoms was 152.7 days. Twenty one of 25 (84%) patients received surgical resection. The mean tumor size was 17.2 cm, with an average mitotic index of 7.6 per 50 high power fields. Thirteen of 14 (92.9%) patients had a high-risk tumor. Five patients were disease-free at a mean followup of 11 months, 2 patients had stable disease and 2 patients had progressive disease, and one patient had a partial response. In conclusion, symptomatic patents have an increased incidence of high-risk tumors and metastases at presentation. Adjuvant therapy with imatinib improves disease-free survival in patients with large abdominal GIST tumors, but no change in overall survival was noted. Finally, GISTs should be considered in the differential diagnosis of an abdominal mass in an elderly patient.

## 1. Introduction

Gastrointestinal stromal tumors (GISTs) are the most common mesenchymal tumors of the gastrointestinal tract. Terminology used to describe gastrointestinal mesenchymal tumors has been variable. Traditionally, these tumors have been called leiomyomas, cellular leiomyomas, or leiomyosarcomas, depending on the degree of cellularity, mitotic activity, and evidence of dissemination, or leiomyoblastomas, when showing epithelioid morphology. Although the term gastrointestinal stromal tumor is now preferred, phenotypic overlap between leiomyomas and GISTs exists, especially in that many GISTs show *α*-smooth muscle actin expression and some show desmin expression [1]. The incidence of GISTs has been historically underestimated prior to the introduction of CD117 staining. The annual worldwide incidence of GISTs since introduction of CD117 staining increased from 1.1 per 100,000 people to 2.1 per 100,000 people. Commensurate with an increased overall incidence has been a 25-fold increase in the age-adjusted incidence of GISTs (from 0.028 per 100,000 in 1992 to 0.688 per 100,000 in 2002), with a current annual incidence of 14.5/100,000 population [2]. Most patients with GISTs are asymptomatic although patients with advanced disease may present with symptoms of a mass lesion, abdominal pain, or bleeding. At least 10 to 30% of GISTs are discovered incidentally during laparotomy, endoscopy, or other imaging studies, with 15% to 50% of GISTs presenting with metastatic disease [3]. GISTs initially presenting as an abdominal mass are exceedingly rare, and only 21 such cases have been reported in the world literature (Table 1). In this paper, we discuss four additional cases of GISTs presenting as an abdominal mass admitted at a tertiary care teaching hospital in New Delhi, India and provide a pertinent review of literature. 

## 2. Case Reports

### 2.1. Case  1

A 60-year male presented with a five-month history of an increasing lower abdominal mass with occasional pain. He denied nausea, vomiting, weight loss, or change in bowel habits. Physical examination revealed a fixed, mass occupying the right lumbar and iliac fossa. Contrast-enhanced computed tomography (CECT) scan of the abdomen and pelvis identified a 25 × 15 cm large necrotic mass arising from the small intestine with a 1 cm hypodense liver lesion. The patient underwent an exploratory laparotomy and resection of the tumor and involved segment of the ileum. No evidence of lymph node or peritoneal metastasis was noted. The histopathological examination of the resected tumor revealed a GIST tumor with free margins. The mitotic index was 5/50 high-power fields (HPFs). The cells showed consistent intracytoplasmic immunoreactivity for CD117 and CD34. The patient was treated with adjuvant imatinib therapy. At one year of the followup, the patient had no evidence of additional metastatic disease, and the liver metastasis had shrunk to 0.5 cm.

### 2.2. Case  2

A 45-year female presented with a three-month history of a progressively enlarging mass in the epigastrium associated with pain and constipation. She denied nausea, vomiting, or weight loss. Physical examination revealed a mobile mass in the periumbilical region partly extending into epigastrium and the right lumbar region. A contrast-enhanced CT scan of the abdomen and pelvis revealed a 15 × 10 cm cystic mass arising from the mesentery of the small bowel. The patient underwent an exploratory laparotomy and was found to have a cystic mass arising from the greater curvature of the stomach. A subtotal gastrectomy with Billroth II gastrojejunostomy was performed. Histopathology of the resected specimen identified a malignant GIST tumor with negative surgical margins and no lymph node involvement. The mitotic index was 7/50 HPF. The cells showed consistent intracytoplasmic immunoreactivity for CD117 and CD34. The patient received adjuvant imatinib therapy and was disease-free at 1-year followup.

### 2.3. Case  3

A 60-year male presented with a 10-month history of an asymptomatic increasing upper abdominal mass. Physical examination revealed a well-defined mobile hard mass in the epigastrium extending to the left hypochondrium and umbilical region. Contrast-enhanced CT scan of the abdomen and pelvis identified a 30 × 10 cm mass arising from the greater curvature of the stomach with no evidence of metastasis. Esophagogastroduodenoscopy (EGD) revealed no mucosal lesions. At laparotomy, the patient was found to have a tumor arising from the posterior wall of the stomach which was adherent to the spleen and pancreas. An *enbloc *subtotal gastrectomy, splenectomy, and distal pancreatectomy were performed. Histopathology revealed a GIST tumor with negative surgical margins. The mitotic index was 9/50 HPF. The cells showed consistent intracytoplasmic immunoreactivity for CD117 and CD34. The patient received adjuvant imatinib therapy and was disease-free at 9-month followup.

### 2.4. Case  4

A 72-year female presented with a 7-month history of a slowly increasing mass in the lower abdomen. She denied nausea, vomiting, weight loss,or change in bowel habits. Physical examination revealed a well-defined, firm, mobile mass occupying the umbilical, hypogastric, and right iliac fossa. The CA-125 tumor marker was normal. Contrast-enhanced CT scan of the abdomen and pelvis identified a 25 × 20 cm lobulated, heterogeneous soft tissue mass with areas of hemorrhage and necrosis. There was no evidence of metastases. The source of the tumor was unclear though a retroperitoneal sarcoma or ovarian carcinoma seemed likely. At laparotomy, a 20 cm mass was identified arising from the gastrocolic ligament and the hepatic flexure of the transverse colon. A right hemicolectomy with ileo transverse anastomosis was performed. Histopathology revealed a GIST tumor with negative surgical margins. The mitotic index was 6/50 HPF. The cells showed consistent intracytoplasmic immunoreactivity for CD117 and CD34. The patient received adjuvant imatinib therapy and remained disease-free at 1-year followup.

## 3. Materials and Methods

A comprehensive English and non-English search for all articles pertinent to GISTs was conducted for the period of 2001 and 2009 using PubMed, a search engine provided by the U.S. National Library of Medicine and the National Institutes of Health. Key words searched included: gastrointestinal stromal tumors, GISTs, and abdominal mass. Cases identified were analyzed according to age and gender of the patients, duration of symptoms, preoperative investigations and diagnosis, tumor size, number of mitotic figures, treatment, and patient outcome. Tumors were classified as very low-, low-, moderate-, and high-risk tumors based on criteria established by Miettinen and Lasota (Table 2) [24]. The response to the therapy was assessed by using the modified computed tomography response evaluation criteria [25]. Data was tabulated, and calculations were performed using Microsoft Excel statistical functions. Statistical analysis included a mean and median tumor size and a number of mitotic figures, and Pearson's coefficient was used to determine the correlation between tumor size and mitotic index.

## 4. Results

Twenty-five cases of GISTs presenting with abdominal mass, including 4 cases discussed here, have been reported in the world literature since 2001. The mean age was 51.2 (17–83) years with an M : F ratio of 1 : 2.1. The mean duration of symptoms was 152.7 days (1–1440 days). All patients (100%) had abdominal pain or abdominal mass at presentation. Contrast-enhanced computer tomography (CECT) was the most commonly used modality of investigation. Preoperative biopsy was done in five patients and fine needle aspiration cytology (FNAC) in two patients. Eight of 25 patients (4 with biopsy, 1 with FNAC, and 3 based on CECT apprearance) had a preoperative diagnosis of GIST, which was confirmed by the histopathology of the resected specimen. In one patient, the biopsy was reported as a leiomyosarcoma. Twenty one of 25 (84%) patients had surgical resection of the tumor, 2 of 25 (8%) patients had an unresectable tumor, one patient refused treatment, and treatment details were not available for additional one patient. The stomach was involved in 9 cases, the small bowel in 8 cases, mesentery in 4 cases, and pancreas and transverse colon in one case each. Primary site of the tumor was not mentioned in two cases. The mean tumor size was 17.2 (4.5–30) cm, and the mitotic index was mentioned in only 14 patients, with an average mitotic index of 7.6 (2–13) per 50 high-power fields. Thirteen of 14 (92.9%) assessable patients had a high-risk tumor, and one patient had a low-risk tumor. Five of 25 (20%) patients had metastases and the most common sites of metastases being peritoneum followed by lymph node and liver. Twelve patients received adjuvant imatinib therapy (Glevac), including two patients with an inoperable tumor. Followup was available for only 2 of 9 patients who had surgery alone. One patient was disease-free at 6 months, and another had stable disease at 6 months. Among patients who received imatinib (*N* = 12), five patients were disease-free at a mean followup of 11 (9–12) months, stable disease or progressive disease was observed in two patients each, one patient had partial response, and response to therapy was not mentioned in two patients.

## 5. Discussion

GISTs can occur at any age although they are more common in adults with a peak incidence in the fifth and sixth decades of life. Approximately 70% of GISTs occur in the stomach, 20 to 30% in the small intestine, and nearly 10% in other parts of gastrointestinal tract, omentum, or mesentry. The exact cell of origin and precise steps in tumorogenesis are not well established however, it appears that these tumors are derived from the interstitial cell of Cajal. Loss of heterozygosity of the *NF1* gene and mutation in the proto-oncogene *c-kit* leading to increased expression of KIT (type III tyrosine kinase receptor) and platelet-derived growth factor receptor-alpha (PDGFRA) are thought to be pivotal [26]. The wild-type KIT receptors appear to signal through the MAP kinase pathway [27] as compared to PI3K-AKT cascade used by *KIT* mutations associated with sporadic GISTs [28]. The later finding may explain the variable response of GISTs to imatinib therapy. 

Microscopically GISTs are classified into: spindle cell type (70%), epitheloid type (20%), and mixed spindle cell and epithelioid cell type. On immunohistochemical staining, 95% are CD117 (c-kit) positive, 70% are CD34, and 40% are stain positive for smooth muscle actin. GISTs spread by the hematogenous route with the liver and peritoneum being the most common sites of metastasis. Rarely, metastases occur to the lung, bones, and lymph nodes. 

A preoperative diagnosis of gastrointestinal stromal tumor (GIST) is difficult given the nonspecific signs and symptoms. Abdominal pain and GI bleeding are the most common presenting complaints. Patients in whom a GIST presents as an abdominal mass are exceedingly rare, and only 25 cases (including 4 cases discussed here) have been reported (Table 1). The 4 cases presented here mimicked other surgical conditions like cecal tumor, mesenteric cyst, stomach carcinoma, ovarian carcinoma, and retroperitoneal tumor both clinically and radiologically. Contrast-enhanced CT scan is the imaging modality of choice for patients with suspected abdominal mass, as it helps in both preoperative staging and to evaluate for metastatic disease. There are no specific CT findings for GIST tumors although they typically appear as an inhomogeneous mass with areas of necrosis and hemorrhage (usually in the center), while viable tumor areas show contrast enhancement (usually at the periphery) [29]. Liver metastasis typically appears as hypodense area, though at times they may have a hyperdense rim. Preoperative biopsy carries a risk of hemorrhage due to the friable nature of these tumors, and hence it is generally avoided if definitive surgery is planned. A fine-needle aspiration may provide adequate tissue to exclude other malignancies and, combined with immunohistochemistry and reverse-transcriptase polymerase chain reaction analysis for *KIT *mutations, will generally confirm the diagnosis of GIST if a biopsy is necessary [30]. Positron emission tomography (PET) scan is generally not used in the evaluation of GISTs although it is helpful in the early detection of tumor response to imatinib therapy and in assessing equivocal metastatic lesions. 

Complete surgical resection is the treatment of choice, and biological therapy (imatinib) is recommended for incomplete resection and unresectable or metastatic disease in patients with primary or recurrent disease. In adult patients with a complete (R0) resection, the FDA has approved adjuvant therapy with imatinib for tumors with *KIT*-positive mutations. Interim results in such patients have shown that imatinib therapy has increased recurrence-free survival for moderate-to-high-risk tumors (tumor size >6 cm), without an improvement in the overall survival [31]. Radiation therapy and chemotherapy have a very limited role in the management of GISTs outside of clinical trials. 

Tumor size and mitotic index are the two most important prognostic factors used for risk stratification of GIST (Table 2). Additional factors such as anatomic location, histologic variant, and type of mutations have also been associated with varying prognoses and differences in overall survival rates [32]. In this paper, we did not identify a definitive correlation between tumor size and mitotic index (*r* = − 0.2). Further, the tumor size and mitotic index did not predict the behavior of the GISTs for this group. These findings indicate that other factors such as type of mutation may have primary influence on the aggressive behavior of GISTs. Finally, Mussi et al. have reported that symptomatic patients have an increased incidence of intermediate- and high-risk tumors as well as metastasis at presentation [33]. In a review of 28 patients with GISTs, they found that 63% of patients were symptomatic and 28.5%, 39%, and 18% of patients had intermediate- and high-risk tumors and metastases, respectively. All patients (100%) discussed here were symptomatic, and the mitotic index was known in 14 patients of whom 7.1%, 85.7%, and 26.3% of patients had low- and high-risk tumors and metastases, respectively.

## 6. Conclusion

Gastrointestinal stromal tumors are highly aggressive tumors, with uncertain etiology. Other than tumor size and mitotic index, additional factors such as the anatomic site, histologic variant, and type of mutation may influence the outcome. There appears to be no correlation between tumor size and mitotic index. Symptomatic patents are noted to have a higher incidence of high-risk tumors and metastases at presentation. Adjuvant therapy with imatinib for patients with intermediate- and high-risk tumors with R0 resection improves disease-free survival with no effect on the overall survival. Finally, GISTs should be considered in the differential diagnosis of an abdominal mass in adult patients.

## Figures and Tables

**Table 1:** Published reports of patients with gastrointestinal stromal tumor presenting as mass per abdomen between 2001 and 2009.

Author, year	Age, sex	Duration of symptoms	Preoperative investigations	Preoperative diagnosis	Treatment	Tumor size, cm	mitosis/50 HPF	Primary site	Metastases	Followup
Johnston et al., 2001 [4]	17, F	1 week	CECT and Biopsy	GIST	CT^II^+ S	NM	NM	Stomach		DF, 11 months
Cheon et al., 2003 [5]	38, M	1 week	CECT	NM	S + CT^I^	10	4	Stomach		DF
Gupta et al., 2004 [6]	18, F	1 week	X-ray abdomen-	SBO/ appendicular lump	S	NM	NM	Transverse colon		DF, 6 months
Froehner et al., 2004 [7]	62, M	6 months	US, CECT and Biopsy	GIST	CT^I^	NM	NM	NM		PD, 3 months
Yeat et al., 2004 [8]	83, F	3-4 days	CECT	Uterine Leiomyosarcoma	S	20	2	Ileum	Peritoneum	LOF, 2 months
	48,F	4 months	CECT	Ovarian cancer	S + CT^I^	20	3	Jejunum	Liver, LN, Peritoneum	No long-term followup
Sinha et al., 2004 [9]	57, M	NM	CECT	GIST	S	15	NM	Mesentery		NM
Arora et al., 2005 [10]	33, F	NM	CECT and Biopsy	GIST	NM	NM	NM	Stomach		NM
Issar et al., 2006 [11]	63, F	5 days	CECT and FNAC	GIST	S	20.4	NM	Stomach		NM
Basile et al., 2006 [12]	52, M	2 weeks	CECT	NM	S + CT^I^	18	>10	Mesentery	Peritoneum	SD, 17 months
Shanmugam et al., 2006 [13]	58, F	2 days	US and CECT	NM	S	6	3	Stomach		NM
Han et al., 2007 [14]	76, F	1 day	CECT	GIST	S + CT^I^	13	13	Small intestine		PD, 18 months^III^
Gupta et al., 2007 [15]	17,F	3 months	CECT and Biopsy	Uterine Leiomyosarcoma	S + CT^I^	30	>5	Mesentery	Cervix, omentum, LN	PD, 4 months
Shetty et al., 2007 [16]	28, M	2 months	CECT	NM	S	17.6	NM	Jejunum		SD, 6 months
Adhikari et al., 2008 [17]	60, F	4 months	CECT	GIST	S	4.5	NM	Duodenum		NM
Saha et al., 2008 [18]	65, F	3 months	CECT	NM	S	11	NM	Stomach		NM
Kale et al, 2008 [19]	61, F	1-year	CECT and Biopsy	GIST	CT^I^	17	NM	Stomach		PR, 12 months
Harindhanavudhi et al., 2009 [20]	63, F	4 years	CECT and FNAC	Hemorrhagic pancreatic cyst	Refused treatment	16	NM	Pancreas		NM
Majdoub Hassani et al., 2009 [21]	54, M	3 months	CECT	NM	S	12	20	Ileocecal junction		NM
Angioli et al., 2009 [22]	38, F	NM	MR imaging	Ovarian cancer	S	17	17	NM		NM
Ulusan et al., 2009 [23]	52, F	Few days	CECT	GIST	S + CT^I^	18	3	Small intestine		NM
Current series	60, M	5 months	CECT	Cecal tumor	S + CT^I^	25	5	Small intestine	Liver	SD, 11 months
	45, F	3 months	CECT	Mesenteric cyst	S + CT^I^	15	7	Stomach		DF, 12 months
	60, M	10 months	CECT	Carcinoma stomach	S + CT^I^	30	9	Stomach		DF, 9 months
	72, F	7 months	CECT	Retroperitoneal sarcoma/Ovarian cancer	S + CT^I^	25	6	Mesentery		DF, 12 months

F: female, M: male, NM: not mentioned, CECT: contrast-enhanced computed tomography scan, SBO: small bowel obstruction, FNAC: fine needle aspiration cytology, S: surgery, HPF: high-power field, NM: not mentioned, MR: magnetic resonance imaging, LN: lymph node, GIST: gastrointestinal stromal tumor, US: ultrasound, CT: chemotherapy, DF: disease-free, PD: progressive disease, SD: stable disease, LOF: lost to follwup.

^I^Adjuvant imatinib therapy.

^II^Two cycles of dacarbazine and doxorubicin.

^III^Patient discontinued imatinib after few months.

**Table 2:** Risk stratification of primary gastrointestinal stromal tumors for aggressive behavior based on tumor size, site, and mitotic index [24].

Tumor parameters		Risk for progressive disease (%), based on site of origin			
Mitotic rate	Size	Stomach	Jejunum/Ileum	Duodenum	Rectum
≤5 per 50 HPF					
	≤2 cm	None (0%)	None (0%)	None (0%)	None (0%)
	>2, ≤5 cm	Very low (1.9%)	Low (4.3%)	Low (8.3%)	Low (8.5%)
	>5, ≤10 cm	Low (3.6%)	Moderate (24%)	Insufficient data	Insufficient data
	>10 cm	Moderate (10%)	High (52%)	High (34%)	High (57%)

>5 per 50 HPF					
	≤2 cm	None	High	Insufficient data	High (54%)
	>2, ≤5 cm	Moderate (16%)	High (73%)	High (50%)	High (52%)
	>5, ≤10 cm	High (55%)	High (85%)	Insufficient data	Insufficient data
	>10 cm	High (86%)	High (90%)	High (86%)	High (71%)

HPF: high-power field.

## References

[1] Miettinen M, Sobin LH, Lasota J (2009). Gastrointestinal stromal tumors presenting as omental masses-a clinicopathologic analysis of 95 cases. *American Journal of Surgical Pathology*.

[2] Samelis GF, Ekmektzoglou KA, Zografos GC (2007). Gastrointestinal stromal tumours: clinical overview, surgery and recent advances in imatinib mesylate therapy. *European Journal of Surgical Oncology*.

[3] Roberts PJ, Eisenberg B (2002). Clinical presentation of gastrointestinal stromal tumors and treatment of operable disease. *European Journal of Cancer*.

[4] Johnston DL, Olson JM, Benjamin DR (2001). Gastrointestinal stromal tumor in a patient with previous neuroblastoma. *Journal of Pediatric Hematology/Oncology*.

[5] Cheon YK, Jung INS, Cho YD (2003). A spontaneously ruptured gastric stromal tumor with cystic degeneration presenting as hemoperitoneum: a case report. *Journal of Korean Medical Science*.

[6] Gupta S, Punia RS, Kaushik R (2004). Gastrointestinal stromal tumour of the colon presenting with intestinal obstruction. *Indian Journal of Cancer*.

[7] Froehner M, Ockert D, Aust DE, Wirth MP, Saeger HD (2004). Gastrointestinal stromal tumor presenting as a scrotal mass. *International Journal of Urology*.

[8] Yeat SK, Chen HJ, Pan HS (2005). Large gastrointestinal stromal tumor mimicking a gynecologic tumor. *Taiwanese Journal of Obstetrics and Gynecology*.

[9] Sinha R, Verma R, Kong A (2004). Mesenteric gastrointestinal stromal tumor in a patient with neurofibromatosis. *American Journal of Roentgenology*.

[10] Arora A, Vaghani M, Shan S (2005). Gastrointestinal stromal tumour of stomach. *Indian Journal of Radiology and Imaging*.

[11] Issar P, Dwivedi MK, Issar SK, Pal RK, Dewanagan L (2006). Malignant gastrointestinal stromal tumor. *Indian Journal of Radiology and Imaging*.

[12] Basile A, Kettenbach J, Mundo E (2006). Erratum: primitive mesenteric gastrointestinal stromal tumor with autonomic nerve/ganglionic differentiation presenting as a huge mass with small synchronous nodules. *European Radiology*.

[13] Shanmugam S, Vijayasekaran D, Marimuthu MG (2006). Gastro intestinal stromal tumor: a case report. *Indian Journal of Radiology and Imaging*.

[14] Han SH, Park SEH, Cho GH (2007). Malignant gastrointestinal stromal tumor in a patient with neurofibromatosis type 1. *The Korean Journal of Internal Medicine*.

[15] Gupta N, Mittal S, Lal N, Misra R, Kumar L, Bhalla S (2007). A rare case of primary mesenteric gastrointestinal stromal tumor with metastasis to the cervix uteri. *World Journal of Surgical Oncology*.

[16] Shetty R, Goel T, Reddy S, Chawla A (2007). GIST as a rare cause of LUTS. *The Internet Journal of Surgery*.

[17] Adhikari S, Ray U, Ray R, Biswas N (2008). Malignant gastrointestinal stromal tumor of the third part of the duodenum presenting as gastric outlet obstruction: a rare presentation. *The Internet Journal of Surgery*.

[18] Saha S, Mahapatara L, Kaur N, Shrivastava UK, Gupta S (2008). Pedunculated gastrointestinal stromal tumor presenting as a mobile intra-abdominal lump. *The Internet Journal of Surgery*.

[19] Kale SS, Sachdev MS, Ismail MK, Davila R, Tombazzi CR (2008). A case and literature review of complicated gastrointestinal stromal tumors. *Gastroenterology and Hepatology*.

[20] Harindhanavudhi T, Tanawuttiwat T, Pyle J, Silva R (2009). Extra-gastrointestinal stromal tumor presenting as hemorrhagic pancreatic cyst diagnosed by EUS-FNA. *Journal of the Pancreas*.

[21] Majdoub Hassani KI, Zahid FZ, Ousadden A, Mazaz K, Taleb KA (2009). Gastrointestinal stromal tumors and shock. *Journal of Emergencies, Trauma and Shock*.

[22] Angioli R, Battista C, Muzii L (2009). A gastrointestinal stromal tumor presenting as a pelvic mass: a case report. *Oncology Reports*.

[23] Ulusan S, Koc Z, Kayaselcuk F (2009). Spontaneously ruptured gastrointestinal stromal tumor with pelvic abscess: a case report and review. *Gastroenterology Research*.

[24] Miettinen M, Lasota J (2006). Gastrointestinal stromal tumors: pathology and prognosis at different sites. *Seminars in Diagnostic Pathology*.

[25] Choi H, Charnsangavej C, Faria SC (2007). Correlation of computed tomography and positron emission tomography in patients with metastatic gastrointestinal stromal tumor treated at a single institution with imatinib mesylate: proposal of new computed tomography response criteria. *Journal of Clinical Oncology*.

[26] Fletcher CDM, Berman JJ, Corless C (2002). Diagnosis of gastrointestinal stromal tumors: a consensus approach. *Human Pathology*.

[27] Chian R, Young S, Danilkovitch-Miagkova A (2001). Phosphatidylinositol 3 kinase contributes to the transformation of hematopoietic cells by the D816V c-Kit mutant. *Blood*.

[28] Corless CL, McGreevey L, Haley A, Town A, Heinrich MC (2002). KIT mutations are common in incidental gastrointestinal stromal tumors one centimeter or less in size. *American Journal of Pathology*.

[29] Da Ronch T, Modesto A, Bazzocchi M (2006). Gastrointestinal stromal tumour: spiral computed tomography features and pathologic correlation. *Radiologia Medica*.

[30] Rader AE, Avery A, Wait CL, McGreevey LS, Faigel D, Heinrich MC (2001). Fine-needle aspiration biopsy diagnosis of gastrointestinal stromal tumors using morphology, immunocytochemistry, and mutational analysis of c-kit. *Cancer*.

[31] Demetri GD, von Mehren M, Antonescu CR (2010). NCCN task force report: update on the management of patients with gastrointestinal stromal tumors. *Journal of the National Comprehensive Cancer Network*.

[32] Fujimoto Y, Nakanishi Y, Yoshimura K, Shimoda T (2003). Clinicopathologic study of primary malignant gastrointestinal stromal tumor of the stomach, with special reference to prognostic factors: analysis of results in 140 surgically resected patients. *Gastric Cancer*.

[33] Mussi C, Schildhaus HU, Gronchi A, Wardelmann E, Hohenberger P (2008). Therapeutic consequences from molecular biology for gastrointestinal stromal tumor patients affected by neurofibromatosis type 1. *Clinical Cancer Research*.

